# Evaluating HPV E6/E7 mRNA expression and genotype prevalence in cervical cytology and biopsy samples from Yunnan Province, China

**DOI:** 10.3332/ecancer.2025.1893

**Published:** 2025-04-17

**Authors:** Yuanyue Li, Xiaomei Wu

**Affiliations:** 1 Department of Gynecology, The First People’s Hospital of Yunnan, Kunming 650032, China; 2 The Affiliated Hospital of Kunming University of Science and Technology, Kunming 650032, Yunnan, China

**Keywords:** HPV mRNA, high-grade lesions, cervical cancer, HR-HPV, LR-HPV

## Abstract

Persistent infection with high-risk human papillomavirus (HR-HPV) is a major contributor to the development of high-grade cervical lesions and invasive cervical cancer. The expression of HR-HPV E6/E7 oncoproteins is a key factor in the progression from preinvasive lesions to cervical cancer, making the detection of E6/E7 mRNA a valuable marker of disease progression. This prospective study evaluated human papillomavirus (HPV) E6/E7 mRNA expression levels and genotype distribution in women referred for colposcopy and biopsy in Yunnan Province, China. Out of 106,245 women undergoing routine checkups, 676 met the inclusion criteria for further analysis. Histological examinations revealed a total of 266 cases of cervicitis, 59 cases of cervical intraepithelial neoplasia (CIN1), 151 cases of CIN2, 84 cases of CIN3, 87 cases of invasive cervical cancer and 29 cases of other conditions, including vaginal intraepithelial neoplasia and warts. The HPV mRNA test demonstrated positivity rates of 47.7% for cervicitis, 52.5% for CIN1, 84.1% for CIN2, 85.7% for CIN3 and 93.1% for invasive cervical cancer. The sensitivity and specificity values for the HPV mRNA test were as follows: for CIN2+, sensitivity was 87.0% and specificity 50.3%; for CIN3+, sensitivity was 89.5% and specificity 40.0%; and for invasive cervical cancer, sensitivity was 93.1% with a specificity of 36.3%. The corresponding positive predictive values were 61.4% for CIN2+, 33.6% for CIN3+ and 17.8% for invasive cervical cancer, while the negative predictive values were 80.9%, 91.8% and 97.3%, respectively. The most common HR-HPV genotypes identified were HPV16 (143 cases), HPV18 (116 cases), HPV52 (78 cases) and HPV58 (69 cases). Age-specific analysis revealed HR-HPV prevalence rates of 56.4% in women aged 18–25, 69.3% in those aged 26–35, 65.4% in the 36–45 age group, 54.2% in women aged 46–59 and 67.9% in those over 60. Remarkably, HPV16, HPV18 and HPV52 were consistently the most prevalent high-risk genotypes across all age groups. These findings highlight the significant burden of HR-HPV infection in Yunnan Province and emphasise the importance of incorporating HPV E6/E7 mRNA testing into cervical cancer screening programs. Given the predominance of HPV 16, 18 and 52, future HPV vaccine formulations should prioritise these genotypes to enhance cervical cancer prevention among Chinese women.

## Introduction

Cervical cancer ranks as the fourth most common cancer among women worldwide [1]. In 2020 alone, it accounted for approximately 604,000 new cases and 342,000 deaths, with nearly 90% of these occurring in low- and middle-income countries [1]. The human papillomavirus (HPV), a non-enveloped double-stranded DNA virus belonging to the Papillomaviridae family, is the primary etiological agent of cervical dysplasia and carcinoma. Infecting the reproductive tract, HPV is implicated in more than 95% of cervical cancer cases globally. The prevention of cervical cancer has become increasingly attainable with the transition from traditional cervical cytology to more sensitive HPV testing methods in many countries, enabling the detection and treatment of precursor lesions before cancer develops [2–4]. The 2014 Bethesda System provides a standardised framework for categorising epithelial squamous cell abnormalities, which is crucial for interpreting cervical cytology results. These abnormalities include high-grade squamous intraepithelial lesions (HSILs), low-grade squamous intraepithelial lesions (LSILs), atypical squamous cells of undetermined significance (ASC-US) and squamous cell carcinoma (SCC) [5]. HSIL encompasses cervical intraepithelial neoplasia (CIN) grades 2 and 3, as well as moderate to severe dysplasia and cervical carcinoma. These conditions are typically characterised by the presence of abnormal cervical epithelial squamous cells [6]. It is often associated with persistent infections of high-risk HPV (HR-HPV) genotypes such as HPV 16 and HPV 18 [7]. HSILs are associated with a higher risk of cancer progression due to the integration of HR-HPV into the host genome, which disrupts the E2 gene. This disruption leads to the overexpression of E6/E7 oncoproteins, which interfere with critical cellular pathways and help the virus evade the host immune response – an essential step in carcinogenesis. In contrast, LSIL typically reflects transient HPV infections with a much lower risk of malignancy, often resolving spontaneously within 2–5 years[8].

Testing for HPV E6/E7 mRNA is more specific than HPV DNA testing for detecting CIN2 lesions [9], and it shows promise in predicting the progression of cervical dysplasia [8]. Among women with abnormal Papanicolaou (Pap) test results and a positive HPV DNA test, the risk of developing CIN2 ranges from 20% to 33% [10]. In primary cervical cancer screening using cytology, equivocal morphological abnormalities – classified as ASC-US – account for 2.7% to 5.8% of all cytological cases in China and 1.8% to 2.8% in Europe [11]. The HPV DNA test boasts an almost 100% negative predictive value (NPV), permitting a screening interval of at least 5 years – longer than the 3-year interval recommended for cytology. This extended interval can significantly reduce both the number of tests performed and associated costs [12]. While colposcopy is essential for confirming CIN3+ lesions in women with abnormal screening results, its low sensitivity for CIN3+ makes it unsuitable as a primary screening tool [13]. Furthermore, Sørbye *et al* [14] found that the risk of developing high-grade lesions (CIN2+) during post-colposcopy follow-up is similar for women with a negative cervical biopsy and those with CIN1. This supports the approach of not treating women with CIN1, similar to those with a positive HPV test but with a normal biopsy [15]. A meta-analysis has indicated that a 5-type HPV mRNA test may offer greater specificity than a 13-type HPV DNA test for triaging women with minor cytological lesions [16]. According to research by Castle *et al* [17], CIN1 does not significantly elevate the risk of developing CIN3 beyond the risk posed by the genotype-specific HPV infection itself. Consequently, CIN1 should not be the primary focus of screening efforts, nor is treatment generally recommended, as it often resolves on its own [17]. These findings underscore the urgent need for large-scale clinical datasets to further elucidate the role of HPV infection and mRNA expression in cervical abnormalities among Chinese women. This study focuses on evaluating HPV mRNA expression levels and genotype distributions in women undergoing cytological and histological assessments in Yunnan Province.

## Materials and methods

### Study design

**Figure 1.**
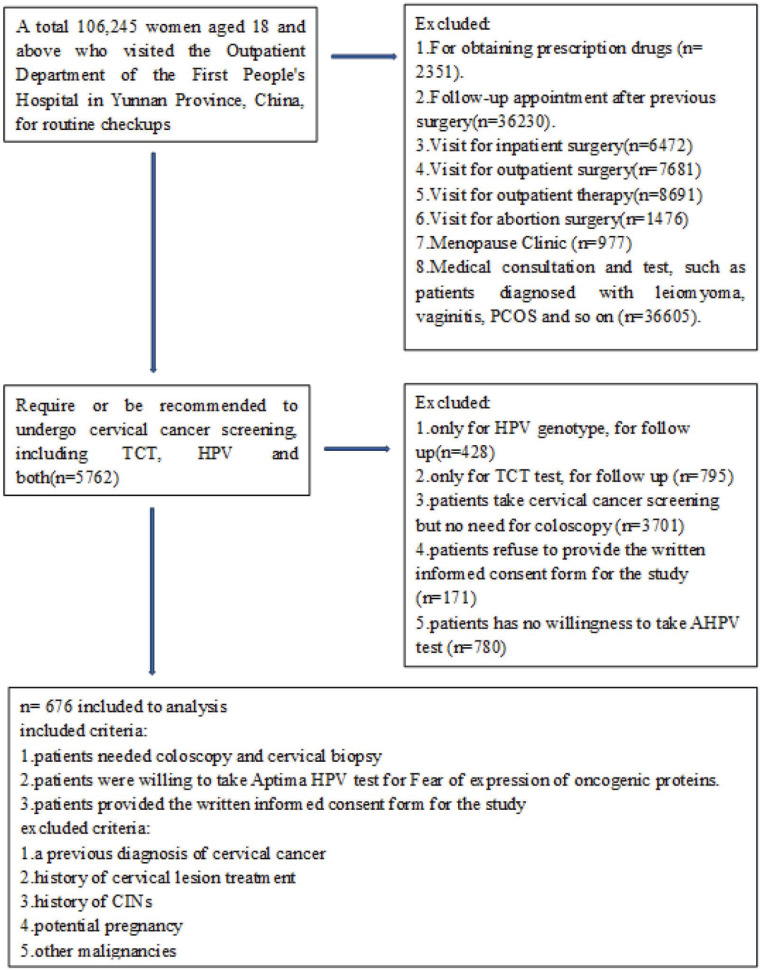
Flowchart.

This prospective study received ethical approval from the Ethics Committee of the Medical Ethics Committee of Yunnan First People’s Hospital (Approval Number: KHLL2023-KY066), located in Kunming, Yunnan Province, China. A total of 106,245 women aged 18 and above, attending routine checkups at the Outpatient Department of Yunnan First People’s Hospital, were screened. Among them, 676 women who met the inclusion criteria were selected for further cytological and histological examinations (Figure 1). Women with a prior diagnosis of cervical cancer, a history of cervical lesion treatment, previous CINs, potential pregnancy or other malignancies were excluded. Written informed consent was obtained from all participants.

### Colposcopy and histological diagnosis

Colposcopy was performed on all participants by a trained gynecologist following the guidelines set by the American Society for Colposcopy and Cervical Pathology. If a participant had a positive genotype test or fluid-based thin-layer cytological test (TCT) result, unsatisfactory colposcopic findings or visible lesions, a combined cervical biopsy with endocervical curettage was carried out. Targeted biopsies were taken from the lesion or aceto-white areas. In cases where no visible lesions or aceto-white areas were observed, a random biopsy was performed at the squamocolumnar junction. Women with no visible lesions and a negative Aptima HPV (AHPV) test did not undergo a biopsy. Histological diagnoses were made by at least two pathologists, following the 2021 WHO Classification of Tumours of the Female Genital Tract, with the P16INK4A immunohistochemistry biomarker used to assist in interpreting CIN2 cases.

### Liquid-based cytology

A single cervical specimen was collected from each participant using a standardised collection device and immediately placed into a ThinPrep container (PreservCyt Solution, Hologic, Inc., MA, USA) for both AHPV testing and liquid-based cytology analysis. Cytology slides were prepared using the automated ThinPrep 2000 machine (Cytyc Corp., MA, USA) and classified according to the 2014 Bethesda System. The categories included: negative for intraepithelial lesions or malignancy (NILM), ASC-US, LSIL, HSIL and SCC.

### Detection of HPV E6/E7 mRNA expression

The AHPV assay was carried out on all liquid-based cytology samples using the automated Panther System (Hologic, Inc., San Diego, CA), which efficiently handled three essential steps: mRNA extraction, amplification and detection. This assay focused on detecting E6/E7 mRNA expression from the fourteen most prevalent high-risk HPV genotypes: HPV16, 18, 31, 33, 35, 39, 45, 51, 52, 56, 58, 59, 66 and 68.

### HPV DNA extraction and genotyping

DNA extraction: HPV genomic DNA was extracted from each cervical specimen using a QIAamp DNA mini kit (Qiagen, Germany), according to the manufacturer’s guidelines. Initially, 1 mL aliquots of the preservative solution containing the cervical cells were centrifuged at 12,000 g for 5 minutes. The supernatant was discarded, and the cell pellet was washed with 500 μL of phosphate-buffered saline. Cells were lysed using 200 μL of 5% Chelex-100 chelating resin for 15 minutes at 100°C. Following lysis, the mixture was centrifuged at 12,000 g for 5 minutes to separate the viral DNA supernatant, which was then retained for further analysis. DNA concentration and purity were assessed using a NanoDrop 2000 spectrophotometer (Thermo Fisher Sci., Waltham, MA). HPV genotyping: The extracted DNA underwent genotyping using the HPV GenoArray test kit (Hybribio CP8304, Chaozhou, China). This kit can identify 15 high-risk HPV genotypes (HPV 16, 18, 31, 33, 35, 39, 45, 51, 52, 53, 56, 58, 59, 66 and 68) and 6 low-risk HPV genotypes (HPV 6, 11, 42, 43, 44 and 81). Polymerase chain reaction (PCR) amplification involved a total reaction volume of 20 μL, comprising 8 μL of DNA template, 10 μL of PCR Master Mix, 0.4 μL of each forward and reverse primer and 0.2 μL of Taq polymerase. The PCR protocol executed in a Veriti thermal cycler (Applied Biosystems, Carlsbad, CA) included an initial denaturation at 95°C for 30 seconds, followed by 35 cycles of denaturation at 94°C for 5 seconds, annealing at 58°C for 30 seconds, extension at 72°C for 30 seconds and a final extension at 72°C for 10 minutes.

### Statistical analysis

Statistical analyses were performed using SPSS software version 26.0 (SPSS Inc., Chicago, IL, USA). The positive predictive value (PPV) and NPV of HPV E6/E7 mRNA expression detection were calculated to assess the diagnostic performance of the test.

## Results

In this study, histological analysis confirmed 266 cases of cervicitis, 59 cases of CIN1, 151 cases of CIN2, 84 cases of CIN3, 87 cases of invasive cervical cancer and 29 cases of other conditions, including vaginal intraepithelial neoplasia and warts. The positivity rates of the

HPV mRNA test varied by condition: 54.5% (170/312) in women with NILM, 66.2% (98/148) in ASCUS/AGCUS cases, 87.0% (47/54) in LSIL cases, 92.8% (64/69) in HSIL cases and 85.9% (61/71) in cases of invasive cervical cancer. In terms of biopsy groups, the positivity rates of the HPV mRNA test were 47.7% (127/266) in cervicitis, 52.5% (31/59) in CIN1, 84.1% (127/151) in CIN2, 85.7% (72/84) in CIN3 and 93.1% (81/87) in invasive cervical cancer. The sensitivity and PPV of the HPV mRNA test were evaluated across various stages of the disease. For CIN2+ lesions, the sensitivity was 87.0%, with a PPV of 61.4%. In CIN3+ lesions, the sensitivity increased to 89.5%, while the PPV decreased to 33.6%. For invasive cervical cancer, the sensitivity was even higher at 93.1%, although the PPV was lower at 17.8%. Detailed information on HPV mRNA expression in women diagnosed with different gynecological conditions can be found in Tables 1 and 2.

The prevalence of HR-HPV, LR-HPV and both single and multiple-genotype infections in women with various grades of cervical lesions and invasive cervical cancer is depicted in the figure. Among the 676 women enrolled, the overall prevalence of HR-HPV and LR-HPV was 57.8% (391/676) and 23.1% (156/676), respectively. In women with cervicitis, the HR-HPV and LR-HPV infection rates were 37.6% (115/266) and 4.5% (7/156), respectively. The HR-HPV positivity rates for CIN1, CIN2 and CIN3 were 62.7% (37/59), 66.2% (100/151) and 61.9% (52/84), respectively, while the LR-HPV positivity rates for CIN1 and CIN2 were 42.3% (113/266) and 24.5% (37/151), respectively. The HR-HPV prevalence rate in women with invasive cervical cancer was 87.4% (76/87). Notably, no LR-HPV was detected in women with CIN3 or invasive cervical cancer. Additionally, the infection types were categorised by genotype: single-genotype infection rates in women with cervicitis, CIN2, CIN3 and invasive cervical cancer were 67.6%, 64.9%, 71.4% and 77.8%, respectively, while multiple genotype infections in these categories were 32.4%, 35.1%, 28.6% and 21.8%, respectively (Table 3).

**Figure 2.**
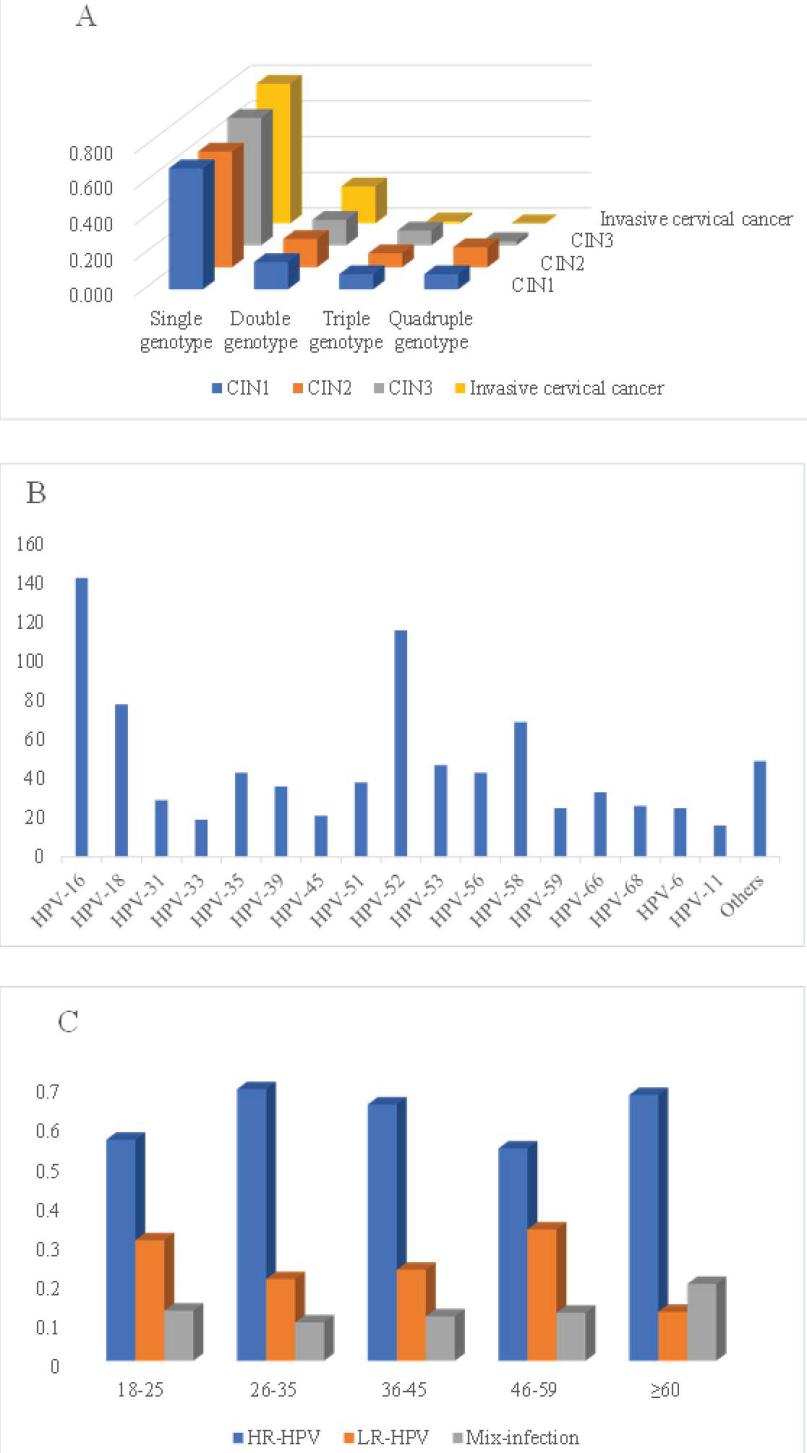
(a): HPV single and multiple genotype infections HPV genotype distributions, (b): HPV genotype distributions, (c): HR-HPV, LR-HPV and other HPV infections among different age groups.

The HPV genotype distribution in single and multiple infections is shown in Figure 2. In this study, HPV16 (143 cases) was the most prevalent genotype, followed by HPV18 (116 cases), HPV52 (78 cases), HPV58 (69 cases), HPV53 (47 cases), HPV56 (43cases) and so on (Figure 2a). In addition, HPV genotype testing revealed 266 single genotypes, 63 double genotypes, 25 triple genotypes and 24 Quadruple genotypes. HPV 16, 18 and 52 were the most common genotypes in various grades of cervical lesions (Figure 2b). Furthermore, the age-specific distribution of HR-HPV, LR-HPV and mixed genotype infections revealed 113 HR-HPVs in women aged 26–35, 121 in the age group 36–45 and 97 in the age group 46–59. 34 LR-HPV genotypes were found in the age groups 26–35, and 43 LR-HPVs were detected in the age groups 36–45. 21 mixed genotypes were discovered in the age group 36–45, 22 in the 46–59 age group and 5 in the 18–25 age group (Figure 2c).

The HPV mRNA test demonstrated positivity rates of 47.7% in cervicitis, with rates progressively increasing by the severity of cervical abnormalities: 52.5% in CIN1, 84.1% in CIN2, 85.7% in CIN3 and 93.1% in invasive cervical cancer. For lesions classified as CIN2+, the sensitivity of the HPV mRNA test was 87.0%, with a PPV of 61.4%. Sensitivity increased further to 89.5% for CIN3+, accompanied by a PPV of 33.6%. The highest sensitivity was observed in cases of invasive cervical cancer, reaching 93.1%, although the PPV was notably lower at 17.8%, as outlined in Tables 4–6.

## Discussion

In this study, HPV mRNA test positivity rates exhibited a clear gradient, increasing with the severity of cervical lesions: 47.7% in cervicitis cases, which showed no evidence of intraepithelial lesions or malignancy; 52.5% in CIN1 cases; 84.1% in CIN2; 85.7% in CIN3; and 93.1% in invasive cervical cancer cases. The sensitivity of the HPV mRNA test was particularly striking at higher disease stages, recorded at 87.0% for CIN2+, 89.5% for CIN3+ and 93.1% for invasive cervical cancer. These sensitivity rates underscore the test’s effectiveness in identifying more advanced and clinically significant disease stages. The PPVs were also notable: 61.4% for CIN2+, 33.6% for CIN3+ and 17.8% for invasive cervical cancer. While the PPVs reflect a strong predictive capacity for high-risk cases, the lower PPV at the most severe stage may indicate the reduced prevalence of invasive cancer within the screening population. This gradient in test performance highlights the potential utility of HPV mRNA testing as a crucial tool in cervical cancer screening and triage protocols. In China, the most prevalent high-risk HPV genotypes associated with cervical cancer are HPV 16, 18, 31 and 33, which is consistent with national prevalence studies that identify HPV 16, 18, 52 and 58 as the most common types [18–20]. Notably, women infected with HPV 16 or HPV 18 face a significantly elevated risk of progressing to high-grade lesions such as CIN2 and cervical cancer. This aligns with existing literature, which indicates that infections with these genotypes substantially increase the likelihood of developing severe cervical pathologies compared to infections with other high-risk types. In contrast, women with ASC-US due to HPV types other than 16 or 18 have a markedly lower risk of progressing to cervical cancer and may not require immediate colposcopic intervention.

Persistent infection with high-risk HPV types, particularly HPV 16, 18 and 45, is a major driver of severe cervical pathologies, including high-grade lesions and SCC. Our findings hold significant implications for public health strategies, especially in regions such as China, where the high prevalence of specific HPV types – most notably HPV 52 (detected in 30 cases) and HPV 16 (in 29 cases) – underscores limitations in current screening approaches and highlights the need for more targeted prevention measures. In this study, we analysed HPV mRNA expression in a cohort of 676 women, examining its prevalence across different age groups. Our results revealed age- and cytology-dependent variations in HPV mRNA positivity rates, consistent with prior studies that report higher HPV prevalence among women with abnormal cytological findings. For example, documented HPV positivity rates of 32.0% in ASC-US cases, 61.0% in LSIL and 71% in HSIL among HPV-positive patients. Similar patterns of HPV distribution have also been observed in large-scale investigations of Chinese populations [21, 22]. The risk of disease progression associated with HPV infection is closely tied to cytological abnormalities. Previous research indicates that approximately 13% of women with LSIL and a positive HPV test progress to CIN2+ during follow-up [23] While spontaneous regression of HPV infections is common – occurring in 28.9% of LSIL and 29.8% of ASC-US cases – persistent infections significantly increase the risk of developing high-grade lesions [24]. Indeed, 10%–20% of women with persistent HPV infections are diagnosed with CIN2+ upon follow-up [24]. Our findings suggest that HPV mRNA testing is an effective tool for risk stratification, particularly in older women diagnosed with CIN3 or invasive cervical cancer. Elevated mRNA positivity rates in women aged 46–59 and ≥60 diagnosed with CIN2, CIN3 and invasive cervical cancer highlight the potential of this approach to improve the identification of high-risk individuals. By incorporating HPV mRNA testing into cervical cancer screening programs, particularly for women with ASC-US cytology, it may be possible to reduce unnecessary colposcopy referrals, enhancing clinical efficiency while optimising resource allocation and patient care.

Our findings further contribute to the ongoing discussion on age-specific screening strategies. Previous research has highlighted challenges associated with HPV DNA screening in younger women (aged 23–39), where high HPV positivity rates are accompanied by a low incidence of cervical cancer. This imbalance can lead to administrative strain and unnecessary treatment. In contrast, HPV mRNA testing, known for its higher specificity, may serve as a more effective primary screening method for women aged 25–33 and 34–69 [14] addressing some of these issues. Overall, our study emphasises the critical role of age-specific and cytology-guided HPV mRNA testing in cervical cancer screening programs. By targeting high-risk populations identified through precise testing methods, we can enhance early detection efforts and significantly reduce the burden of cervical cancer among Chinese women.

## Conclusion

This study reveals a high prevalence of HPV among a referral population in Yunnan Province, with HPV 16, 18, 52 and 58 emerging as the dominant genotypes. These findings are crucial for improving triage strategies for women with atypical ASC-US and other gynecological conditions, paving the way for more precise and effective interventions. The results suggest that HPV testing protocols in China should be further refined to enhance their specificity and improve cervical cancer screening outcomes. Given the prominence of HPV 16, 18, 52 and 58, prioritising these genotypes in the development of next-generation HPV vaccines is imperative for advancing cervical cancer prevention efforts among Chinese women. Continued research is essential to refine triage and vaccination strategies, ensuring they are tailored to the unique epidemiological profile of HPV in this region.

## Conflicts of interest

All authors declare that no conflicts of interest exist.

## Consent for publication

Not applicable.

## Availability of data and materials

The datasets generated and analysed during the current study are not publicly available due to the risk of compromising the individual privacy of participants.

## Figures and Tables

**Table 1.** HPV E6/E7 mRNA expression in patients with different cervical lesions and cancer grades by TCT test.

Disease status	Total	mRNA+ (%)	mRNA- (%)
NILM	312	170 (54.5)	142 (45.5)
ASCUS/AGCUS	148	98 (66.2)	50 (33.8)
LSIL	54	47 (87.0)	7 (12.9)
HSIL	69	64 (92.8)	5 (7.2)
Invasive cervical cancer	71	61 (85.9)	10 (14.0)
Others	22	16 (72.7)	6 (27.3)
Total	676	456 (66.5)	220 (32.5)

NILM = Negative for intraepithelial lesions or malignancy

ASCUS: atypical squamous cells of undetermined significance

LSIL: low-grade squamous intraepithelial lesion

HSIL: high-grade squamous intraepithelial lesion

**Table 2.** HPV E6/E7 mRNA expression in patients with different cervical lesions and cancer grades by biopsy.

Disease status	Total	mRNA+ (%)	mRNA- (%)
Cervicitis	266	127 (47.7)	139 (52.3)
CIN1	59	31 (52.5)	28 (47.5)
CIN2	151	127 (84.1)	24 (15.9)
CIN3	84	72 (85.7)	12 (14.3)
Invasive cervical cancer	87	81 (93.1)	6 (6.9)
Others (eg warts, VIN, VC)	29	18 (62.1)	11 (37.9)
Total	676	456 (66.5)	220 (35.5)

CIN: cervical intraepithelial neoplasia, VIN = Vaginal intraepithelial neoplasia, VC= Vaginal cancer

**Table 3.** HPV mRNA detection rates in women with single versus multiple infections across various cervical conditions (*n* = 676).

Positive	HPV+	Infection types	
		Single infection	Multiple infections
CIN1	59	40 (67.6)	19 (32.4)
CIN2	151	98 (64.9)	53 (35.1)
CIN3	84	60 (71.4)	24 (28.6)
Invasive cervical cancer	87	68 (77.8)	19 (21.8)
Total	410	266 (71.5)	115 (28.5)

**Table 4.** The HPV mRNA test performance in CIN2.

AHPV	biopsy		Total
	CIN2+	Cin1-	
mRNA+	280	176	456
mRNA-	42	178	220
Total	322	354	676

Sensitivity:87.0%, Specificity:50.3%, PPV:61.4%, NPV:80.9%

**Table 5.** The HPV mRNA test performance in CIN3.

AHPV	Biopsy		Total
	CIN3+	CIN2-	
mRNA+	153	303	456
mRNA-	18	202	220
Total	171	505	676

Sensitivity:89.5%, Specificity:40.0%, PPV:33.6%, NPV:91.8%

**Table 6.** The HPV mRNA test performance in invasive cervical cancer.

AHPV	Biopsy		Total
	Invasive cervical cancer	No cancer	
mRNA+	81	375	456
mRNA-	6	214	220
Total	87	589	676

Sensitivity:93.1%, Specificity:36.3%, PPV:17.8%, NPV:97.3%
